# Development of a microarray for simultaneous detection and differentiation of different tospoviruses that are serologically related to *Tomato spotted wilt virus*

**DOI:** 10.1186/s12985-016-0669-1

**Published:** 2017-01-10

**Authors:** Lu-Yuan Liu, He-Yi Ye, Tsang-Hai Chen, Tsung-Chi Chen

**Affiliations:** 1 Department of Plant Industry, National Pingtung University of Science and Technology, Pingtung, 91201 Taiwan; 2 Department of Biotechnology, Asia University, Wufeng, Taichung, 41354 Taiwan; 3 Department of Plant Medicine, National Pingtung University of Science and Technology, Pingtung, 91201 Taiwan; 4 Department of Medical Research, China Medical University Hospital, China Medical University, Taichung, 40402 Taiwan

**Keywords:** *Tospovirus*, N gene, Detection, Identification, Microarray

## Abstract

**Background:**

Tospoviruses, the plant-infecting genus in the family *Bunyaviridae*, are thrips borne and cause severe agricultural losses worldwide. Based on the serological relationships of the structural nucleocapsid protein (NP), the current tospoviruses are divided into six serogroups. The use of NP-antisera is convenient for virus detection, but it is insufficient to identify virus species grouped in a serogroup due to the serological cross-reaction. Alternatively, virus species can be identified by the N gene amplification using specific primers. *Tomato spotted wilt virus* (TSWV) is the type species of the genus *Tospovirus* and one of the most destructive plant viruses. Eight known tospoviruses, Alstroemeria necrotic streak virus (ANSV), Chrysanthemum stem necrosis virus (CSNV), *Groundnut ringspot virus* (GRSV), *Impatiens necrotic spot virus* (INSV), Melon severe mosaic virus (MeSMV), Pepper necrotic spot virus (PNSV), *Tomato chlorotic spot virus* (TCSV) and *Zucchini lethal chlorosis virus* (ZLCV), sharing serological relatedness with TSWV in NP, are grouped in the TSWV serogroup. Most of the TSWV-serogroup viruses prevail in Europe and America. An efficient diagnostic method is necessary for inspecting these tospoviruses in Asia, including Taiwan.

**Methods:**

A microarray platform was developed for simultaneous detection and identification of TSWV-serogroup tospoviruses. Total RNAs extracted from *Chenopodium quinoa* leaves separately inoculated with ANSV, CSNV, GRSV, INSV, TCSV and TSWV were used for testing purposes. The 5’-biotinylated degenerate forward and reverse primers were designed from the consensus sequences of N genes of TSWV-serogroup tospoviruses for reverse transcription-polymerase chain reaction (RT-PCR) amplification. Virus-specific oligonucleotide probes were spotted on the surface of polyvinyl chloride (PVC) chips to hybridize with PCR products. The hybridization signals were visualized by hydrolysis of NBT/BCIP with streptavidine-conjugated alkaline phosphatase. The microarray was further applied to diagnose virus infection in field crop samples.

**Results:**

Amplicons of approximately 0.46 kb were amplified from all tested TSWV-serogroup tospoviruses by RT-PCR using the degenerate primer pair Pr-dTS-f/Pr-dTS-r. Virus species were identified on chips by hybridization of PCR products with respective virus-specific probes. The microarray was successfully used to diagnose TSWV infection in field pepper samples.

**Conclusions:**

In this study, a rapid, sensitive and precise microarray method has been developed to simultaneously detect and identify six TSWV-serogroup tospoviruses. The microarray platform provides a great potential to explore tospoviruses that can help researchers and quarantine staff to prevent invasions of tospoviruses.

**Electronic supplementary material:**

The online version of this article (doi:10.1186/s12985-016-0669-1) contains supplementary material, which is available to authorized users.

## Background

Tospoviruses, classified in the family *Bunyaviridae*, are destructive plant viruses with a very broad host range, infecting more than 1090 plant species, causing lethal necrotic lesions, wilting and dieback of infected plants [1–3]. *Tomato spotted wilt virus* (TSWV) is the first tospovirus found in Australia in 1915 [4, 5]. To date, it is still the most important tospovirus causing severe damage of economically important crops, including tobacco, tomatoes, pepper, cucurbits, lettuce, groundnuts and potatoes throughout Africa, America, Asia, Australasia and Europe [3].

Virions of tospoviruses are enveloped quasi-spherical particles measuring 80–120 nm in diameter, possessing a tripartite segmented ssRNA genome, named large (L), medium (M) and small (S). The L RNA is of negative sense, whereas the M and S RNAs are ambisense [6]. L RNA contains a large open reading frame (ORF) in the viral complementary (vc) strand for encoding an RNA-dependent RNA polymerase (RdRp) [7, 8]. Each of the M RNA and S RNA contains two ORFs separated by an A-U-rich intergenic region (IGR). The M RNA encodes a movement protein NSm from the viral (v) strand [9–12] and the enveloped Gn and Gc glycoproteins from the vc strand [13, 14]. The S RNA encodes an RNA-silencing suppressor NSs protein from the v strand [15, 16] and the RNA-associated nucleocapsid protein (NP) from the vc strand [17].

NP is abundant in infected plant cells [17]. The identity and serology of NP are the most important criteria for identification of tospoviruses. A threshold of 90% amino acid (aa) identity of the NP is proposed to classify tospoviruses at the species level [6, 18]. Currently, 29 tospovirus species are identified based on the sequence determination of the N gene or S RNA [3, 19–24]. In addition, most of the known tospoviruses are clustered into three major serogroups, which are assigned by the TSWV, *Watermelon silver mottle virus* (WSMoV) and *Iris yellow spot virus* (IYSV) due to the serological relatedness of their NPs [25]. The members of the TSWV serogroup principally occur in America and Europe. Alternatively, the tospoviruses belonging to the WSMoV and IYSV serogroups are prevalent in Asian countries [3]. The tospoviruses Alstroemeria necrotic streak virus (ANSV), Chrysanthemum stem necrosis virus (CSNV), *Groundnut ringspot virus* (GRSV), *Impatiens necrotic spot virus* (INSV), Melon severe mosaic virus (MeSMV), Pepper necrotic spot virus (PNSV), *Tomato chlorotic spot virus* (TCSV) and *Zucchini lethal chlorosis virus* (ZLCV) were grouped in the TSWV serogroup [26–28], and they are quarantine viruses in Taiwan and other Asian countries.

Prompt and accurate methods for detection and identification of tospoviruses are crucial for preventing epidemics of domestic and invading species. The serological enzyme-linked immunosorbent assay (ELISA) is the most convenient method for virus detection. Antisera against the NPs of tospoviruses are commonly used to detect tospoviruses. Due to the cross reactions of different species in the same serogroup, the use of NP antisera for virus identification could be limited [28, 29]. The nucleic acid-based reverse transcription-polymerase chain reaction (RT-PCR) using N gene-specific primers can be conducted to identify tospovirus species. However, a single species-specific primer pair for diagnosis of tospoviruses in a farm is insufficient due to the complex virus categories which occurred in a crop or area. Degenerate primers designed from the conserved regions of genomic sequences simplify detection of tospoviruses in one tube-based RT-PCR, but they are inconvenient to identify virus species [30, 31]. The microarray is an efficient method for simultaneous detection and identification of various plant viruses in one assay to solve these problems [32].

In this study, an oligonucleotide-based microarray method was developed for detection and identification of tospoviruses, which are serologically indistinguishable with TSWV. The degenerate primer pair Pr-dTS-f/Pr-dTS-r was designed from the consensus sequences of N genes of TSWV-serogroup tospoviruses and successfully used to amplify a certain cDNA fragment from the total RNAs of *Chenopodium quinoa* leaves separately inoculated with ANSV, CSNV, GRSV, INSV, TCSV and TSWV in RT-PCR. Virus species were further identified by hybridization of the PCR products with the virus-specific oligonucleotide probes that were pre-spotted on the surface of polyvinyl chloride (PVC) microchips. The signals were visualized by hydrolysis of NBT/BCIP with streptavidine-conjugated alkaline phosphatase. Furthermore, the microarray was used to diagnose TSWV infections in field pepper samples. The developed microarray has a great potential to prompt the diagnosis of TSWV-serogroup tospoviruses.

## Methods

### Virus sources

The NY isolate of TSWV was collected from tomato in New York, the United States [30]. The original isolate of GRSV-BR was collected from tomato in Brazil [33]. The BR-03 isolate of TCSV was isolated from tomato in Brazil [34]. INSV-M was isolated from impatiens in the United States [35]. The TcCh07A isolate of CSNV was collected from chrysanthemum in Tochigi Prefecture, Japan [36]. The original isolate of ANSV was collected from *Alstroemeria* sp. in Colombia [27]. All virus cultures were maintained in the local lesion host *Chenopodium quinoa* Willd. by mechanical transmission in an isolated temperature-controlled (25–28 °C) greenhouse according to quarantine regulations. A buffer consisting of 0.01 M potassium phosphate (pH 7.0) and 0.01 M sodium sulfite was used for inoculation.

### Construction of N genes

The sequences of the N genes or S RNAs of tospoviruses were obtained from the GenBank of National Center for Biotechnology Information (NCBI) (www.ncbi.nlm.nih.gov) (Table 1) for design of N gene-specific primer pairs (Table 2). One hundred milligram of plant leaf tissues inoculated with ANSV, CSNV-TcCh07A, GRSV-BR, INSV-M and TCSV-BR-03 were ground to fine powder in liquid nitrogen and placed in microcentrifuge tubes for isolation of total RNAs using the Plant Total RNA Miniprep Kit (GeneMark, GMbiolab Co., Ltd., Taichung, Taiwan) following the manufacturer’s instructions. The complete N gene sequences of tospoviruses were amplified from the aforementioned total RNAs with individual primer pairs (Table 2) using the One-Step RT-PCR Kit (GeneMark). Ten microgram of total RNA, 200 nM individual primers, 25 U One-Step RT-PCR enzyme mix, 1/5 volume of reaction buffer and 1/5 volume of enhancer buffer (GeneMark) were mixed for amplification. Synthesis of cDNAs was conducted at 50 °C for 30 min, and inactivation at 94 °C for 2 min. PCR was performed by 35 cycles of strand separation at 94 °C for 1 min, annealing at 58 °C for 30 s and synthesis at 72 °C for 1 min; and a final reaction at 72 °C for 7 min. The amplicons were ligated with the TOPO TA cloning vector pCR2.1-TOPO (Invitrogen, Carlsbad, CA), and then transferred into the DH5α competent cells following the manufacturer’s instructions. The resultant plasmids named pTOPO-ANSV-N for ANSV, pTOPO-CSNV-N for CSNV-TcCh07A, pTOPO-GRSV-N for GRSV-BR, pTOPO-INSV-N for INSV-M and pTOPO-TCSV-N for TCSV-BR-03 were obtained by the Plasmid Miniprep Purification Kit (GeneMark) following the standard protocols. The nucleotide sequences of all cloned N genes were verified by sequencing using ABI3730 XL DNA Analyzer (Perkin-Elmer Applied Biosysterms, Foster City, CA), performed by Mission Biotech Company (Taipei, Taiwan). The plasmid pTOPO-TSWV-N for TSWV-NY was constructed in the previous study [28].

**Table 1:** Accession codes of N genes or S RNAs used for multiple alignments

Species	Abbreviation	Isolate/Country	Accession code
Alstroemeria necrotic streak virus	ANSV	Original/Colombia	GQ478668
Chrysanthemum stem necrosis virus	CSNV	Original/Brazil	AF067068
*Groundnut ringspot virus*	GRSV	Cb/Brazil	AF251271
*Impatiens necrotic spot virus*	INSV	J/Japan	AB109100
Melon severe mosaic virus	MeSMV	VE440/Mexico	EU275149
Pepper necrotic spot virus	PNSV	T2/Peru	HE584762
*Tomato chlorotic spot virus*	TCSV	10-10-14/USA	KP172480
*Tomato spotted wilt virus*	TSWV	Ordinary/Japan	AB010997
*Zucchini lethal chlorosis virus*	ZLCV	Original/Brazil	AF067069

^a^Italic typing represents official species and standard typing represents tentative species

**Table 2:** The primer pairs used for cloning of N genes of tospoviruses

Virus	Primer name	Sequence (5’ → 3’)
ANSV	ANSV-Sph-Nde-N	GGCATGCGGCATATGTCTAAGGCTAAGTTGACAAGGGAA
	ANSV-Kpn-Xho-Nc	GCTCGAGTTAGGTACCAGCAACACCTGAAATTTTGGATTC
CSNV	CSN152Nco	GCCATGGCTAAAGTTAAGCTTACAAAGG
	CSN932cXho	GCTCGAGAACAAGATCTTTAGGAATAAG
GRSV	GR-Nc-N-2122	GCCATGGCTAAGGTCAAGCTCACA
	GR-Xh-N-2895c	GCTCGAGTGCAACAACAGCAATCTT
INSV	IN2843cS	GGGCATGCATGAACAAAGCAAAGATTACCAAG
	IN2058K	AAGGTACCAATAGAATCATTTTTCCCAAAATC
TCSV	TC-Nc-N-f	GCCATGGCTAAGGTCAAGCTCACC
	TC-Xh-N-c	GCTCGAGTGCAACACCTGAAATTTT
TSWV	TN1990Xho	GCTCGAGAGCAAGTTCTGCGAGTTTTGC
	TN2763Ncoc	GCCATGGCTAAGGTTAAGCTCACTAAG

### Design of degenerate primers and virus-specific probes

The Lasergene 7 software package (DNASTAR, Madison, WI, USA) was used to analyze sequences. Multiple alignments of nucleotide sequences of N genes listed in Table 1 were conducted by the MegAlin program. The conserved regions of N genes were used to design degenerate primers, and the virus-specific probes were designed from the variable regions of N genes.

### Amplification assay for degenerate primers

PCR was conducted to evaluate the degenerate primers using the constructed plasmids. The reaction mixture of 25 μl is comprised of 100 ng of plasmid DNA, 2.5 μl of 10× Taq buffer (50 mM Tris–HCl, pH 8.0, 1 mM EDTA, 1 mM DTT and 50% glycerol) (Protech, Taipei, Taiwan), 1 μl of 10 mM dNTPs (Protech), 1 μl of 100 μM forward primer, 1 μl of 100 μM reverse primer and 0.1 μl of Pro Plus Taq DNA polymerase (5 U/μl) (Protech). The amplification was carried out by Applied Biosystems GeneAMP PCR System 9700 (Thermo Fisher Scientific Inc., MA, USA) under the setting of a hot start at 95 °C for 5 min; 35 cycles of 95 °C for 30 s, 50 °C for 40 s and 72 °C for 30 s; and a final reaction at 72 °C for 6 min. The amplicons were visualized by 2% agarose gel electrophoresis and ethidium bromide (EtBr) staining. For virus detection, the mixture of 25 μl consisting of 200 ng of plant total RNA, 0.2 μl of M-MuLV reverse transcriptase (5 U/μl) (Protech), 2.5 μl of 10× Taq buffer (Protech), 1 μl of 10 mM dNTPs (Protech), 0.1 μl of 100 μM forward primer, 0.1 μl of 100 μM reverse primer and 0.1 μl of Pro Plus Taq DNA polymerase (5 U/μl) (Protech) was used in RT-PCR amplification. Complementary DNA was synthesized at 45 °C for 30 min then termination at 95 °C for 5 min. The PCR amplification was set as a hot start at 95 °C for 5 min; 35 cycles of 95 °C for 30 s, 50 °C for 40 s and 72 °C for 30 s; and a final reaction at 72 °C for 6 min. The amplicons were observed by 2% agarose gel electrophoresis and EtBr staining.

### Microarray preparation and assay

Twenty micromolar of each probe were spotted on the surface of PVC microchips with an automatic spotting machine (DR. Chip Biotechnology Inc., Miaoli, Taiwan) and immobilized by an ultraviolet crosslinker (UV Light Enterprise Co., Taichung, Taiwan) with 1.2 J for 5 min following the previous report [37]. In addition to the spots for specific tospoviruses, 100 μM of the commercial probe Anti-VP1 (Dr. Chip) was spotted for positive control of hybridization. Ten microliter of PCR products were mixed with 200 μl of commercial DR. Chip Hyb™ buffer (Dr. Chip) containing the 5’ end-biotinylated oligonucleotide (VP1) complementary to the sequence of positive control probe Anti-VP1. The mixture was denatured in boiling water for 5 min then immediately chilled on ice for 3 min. Subsequently, the mixture was transferred into the chip chamber and the chips were incubated at 50 °C in a commercial hybridization oven (DR. Chip) with vibration for 1 h. After hybridization, the chips were washed by 200 μl of Wash buffer™ (Dr. Chip) in a 1 min incubation for 3 repeats. Then, the chip chamber was inverted and tapped on a paper towel to remove the residual liquid. Thereafter, the mixture of 0.2 μl of DR. Chip Srep-AP™ (Streptavidin-conjugated alkaline phosphatase, Dr. Chip) in 200 μl of DR. Chip Block Reagent™ (Dr. Chip) was transferred into the chip chamber to allow a 30 min incubation at room temperature. The chamber was washed 3 times with 200 μl of wash buffer as described above, followed by rinsing the chips with 200 μl of Detection Buffer™ (Dr. Chip). After discarding the liquid, 4 μl of DR. NBT/BCIP™ (nitro blue tetrazolium/5-bromo-4-chloro-3-indolyl phosphate, Dr. Chip) was mixed with 196 μl of detection buffer, and the mixture was added to the hybridization chamber. The colorimetric development was allowed to react in the dark for 10 min at room temperature, and then the detection liquid was discarded. The chip was rinsed with deionized water twice, and the developed patterns were recorded by DR. Scanning Reader (Dr. Chip).

### Establishment of the detection platform of the microarray

The PCR product amplified from pTOPO-TSWV-N [28] using the designed primers was serially diluted for testing the detection sensitivity of the microarray. Total RNAs extracted from TSWV-infected pepper samples were used for testing the virus identification by microarray. The protocols of the microarray were described above.

### Indirect enzyme-linked immunosorbent assay (ELISA)

Indirect ELISA was conducted to detect TSWV in diseased plant samples according to the previous report [38]. The crude extracts of plant samples at a 1/20 dilution with coating buffer (0.05 M sodium carbonate, pH 9.6, containing 0.01% sodium azide) were used for assays. The monoclonal antibody against the NSs protein of TSWV, MAb-TNSs-82D3B4 [39], diluted with conjugate buffer (PBST containing 2% PVP-40 and 0.2% BSA), was used at a 1/8000 dilution. The alkaline phosphatase (AP)-conjugated goat anti-mouse IgG (Jackson ImmunoResearch Laboratories) was used at a 1/5000 dilution as the secondary antibody. The AP substrate tablets (ρ-Nitrophenyl phosphate disodium salt hexahydrate) (Sigma-Aldrich, St. Louis, MO) dissolved in substrate buffer (9.7% diethanolamine and 0.02% NaN_3_, pH 9.8) were used for colour development. The absorbance at 405 nm (A_405_) was determined by Model 680 microplate reader (Bio-Rad) after 10 to 60 min of substrate addition.

## Results

### Detection test of the designed degenerate primers and virus-specific probes in the microarray

**Fig. 1:**
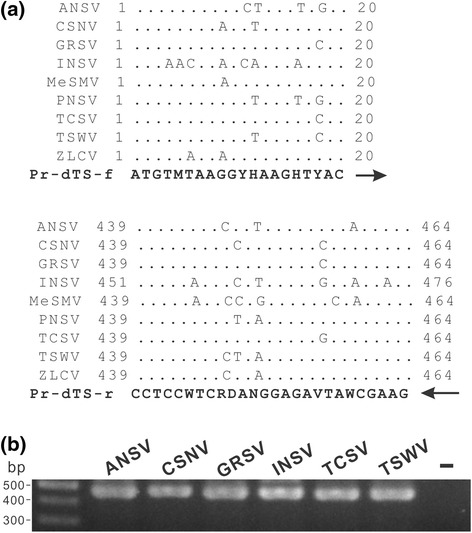
Design of degenerate primers for detection of the TSWV-serogroup tospoviruses. **a** Multiple alignments of the N gene sequences of ANSV, CSNV, GRSV, INSV, MeSMV, PNSV, TCSV, TSWV and ZLCV. The accession codes of individual sequences and virus abbreviations are listed in Table 1. The sequences of forward primer Pr-dTS-f and reverse primer Pr-dTS-r are shown in *bold*, and their corresponding positions of N gene sequences of individual tospoviruses are presented. The directions of primers (5’ to 3’) are indicated by *arrows*. **b** The primer pair Pr-dTS-f/Pr-dTS-r was used in a polymerase chain reaction for amplification of N gene fragments from the plasmids carrying the N gene sequences of ANSV, CSNV, GRSV, INSV, TCSV or TSWV. “-” represents a blank control

**Fig. 2:**
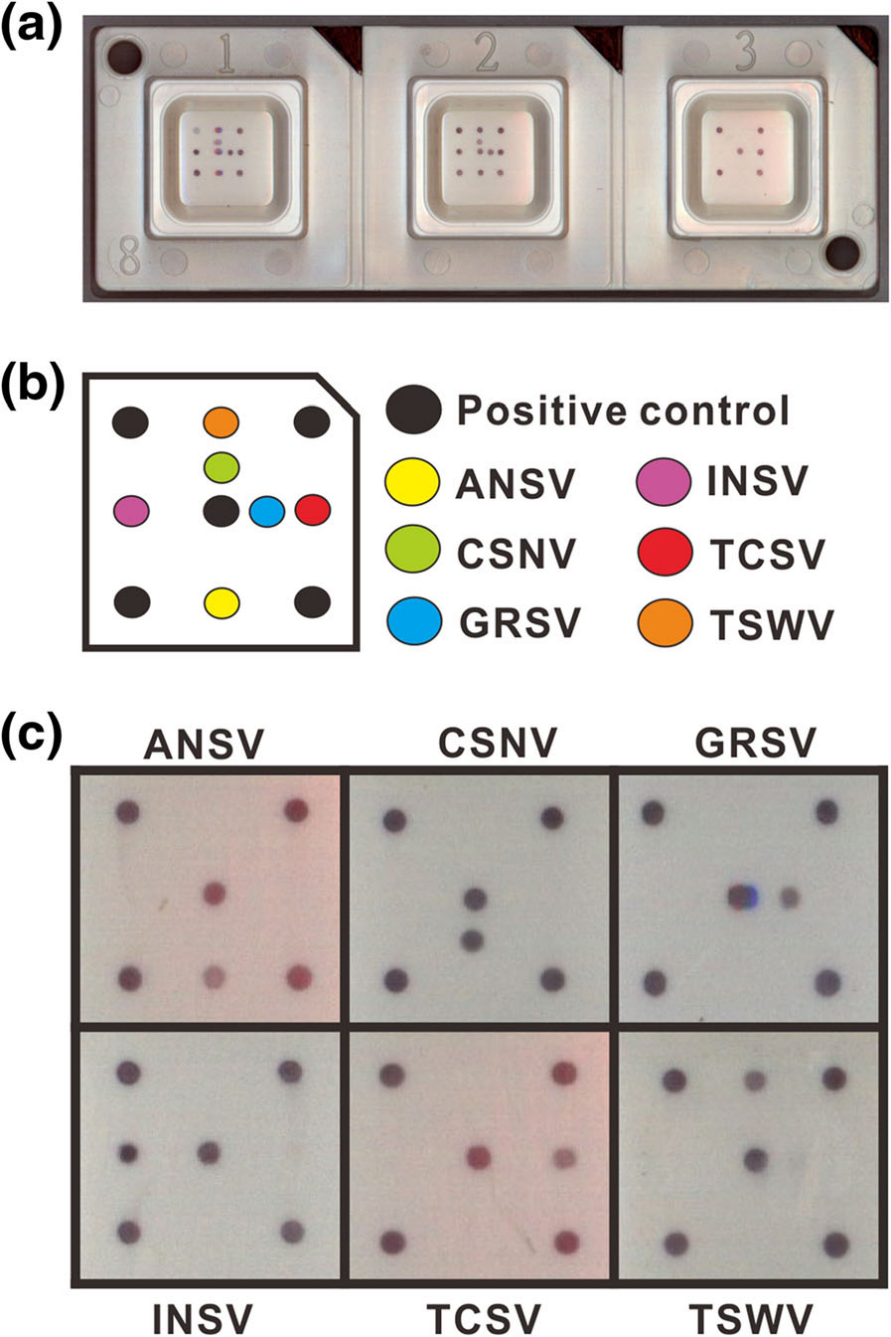
Establishment of the microarray. **a** The biochip device. **b** The graph represents the biochip elements. The positions of virus-specific probes spotted on the surface of biochips are indicated by *color spots*. **c** Hybridization patterns of individual tospoviruses. The degenerate primer pair Pr-dTS-f/Pr-dTS-r was used in a polymerase chain reaction for amplification of N gene fragments from the plasmids carrying the N gene sequences of ANSV, CSNV, GRSV, INSV, TCSV or TSWV

Two conserved regions located at the first 20 nucleotides and middle portion of N genes of the TSWV-serogroup tospoviruses were used to design the forward primer Pr-dTS-f and reverse primer Pr-dTS-r, respectively (Fig. 1a). Both primers were synthesized with a 5’-biotinylation (Table 3). The primer pair Pr-dTS-f/Pr-dTS-r was able to amplify a DNA fragment of approximately 0.46 kb from all tested plasmids carrying the N genes of ANSV, CSNV, GRSV, INSV, TCSV or TSWV in PCR (Fig. 1b). The virus-specific probes designed from the N genes of ANSV, CSNV, GRSV, INSV, TCSV and TSWV were synthesized with an addition of 20 thymidine deoxynucleosides (T_20_) at the 5’ terminus for fixing on microchips (Table 3). The spotted positions of individual probes in the microarray are indicated in Fig. 2a and b. The PCR products amplified from different plasmids were incubated with the microarray for hybridization. Colorization of the specific spots indicated that the virus species can be identified by the microarray (Fig. 2c).

**Table 3:** Primers and probes used for the microarray

Primer/Probe	Length (bp)	Sequence (5’ → 3’)	Specificity
Primer name			
Pr-dTS-f	20	Biotin ATGTMTAAGGYHAAGHTYAC	TSWV serogroup
Pr-dTS-r	26	Biotin GAAGCWATVAGAGGNADRCTWCCTCC	
Probe name			
Pb-AN	40	T_20_ ATGGACTTCCTTTGAATGAT	ANSV
Pb-CS	40	T_20_ ACGGGCTTAGCTTGAATGAT	CSNV
Pb-GR	40	T_20_ ACGGGCTGCCTCTGGCAGAT	GRSV
Pb-IN	40	T_20_ ATGGTCTTGCAACCACAGAT	INSV
Pb-TS	40	T_20_ ATGGATTACCTCTCGATGAT	TSWV

### Sensitivity assay of microarray

**Fig. 3:**
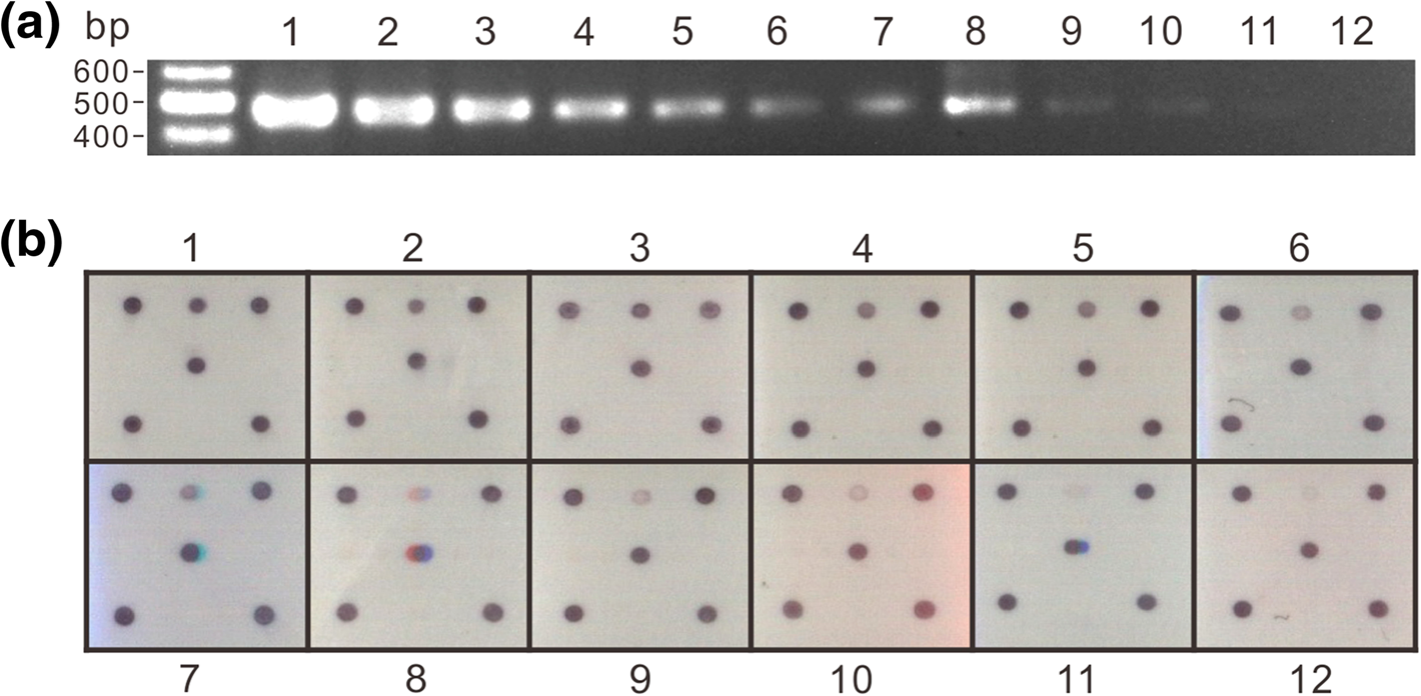
Sensitivity assay of the microarray. The PCR product amplified from pTOPO-TSWV-N using the primer pair Pr-dTS-f/Pr-dTS-r was diluted as shown for the test. **a** The diluted amplicons were analysed by agarose gel electrophoresis with ethidium bromide staining. **b** The microarray result. The concentrations of amplicon are indicated as 1: 250 ng; 2: 125 ng; 3: 62.5 ng; 4: 32 ng; 5: 16 ng; 6: 8 ng; 7: 4 ng; 8: 2 ng; 9: 1 ng; 10: 0.5 ng; 11: 0.2 ng; and 12: 0.1 ng

The PCR product amplified from the plasmid pTOPO-TSWV-N was serially diluted, initiating from 250 to 0.1 ng, to determine the sensitivity of microarray detection. EtBr staining in agarose gel electrophoresis was performed for comparison. The results showed that the microarray was able to detect a lesser amount of 0.1 ng of the PCR product that is more sensitive than EtBr staining with a detection limitation of 0.2 ng of the PCR product (Fig. 3).

### Microarray in virus detection

**Fig. 4:**
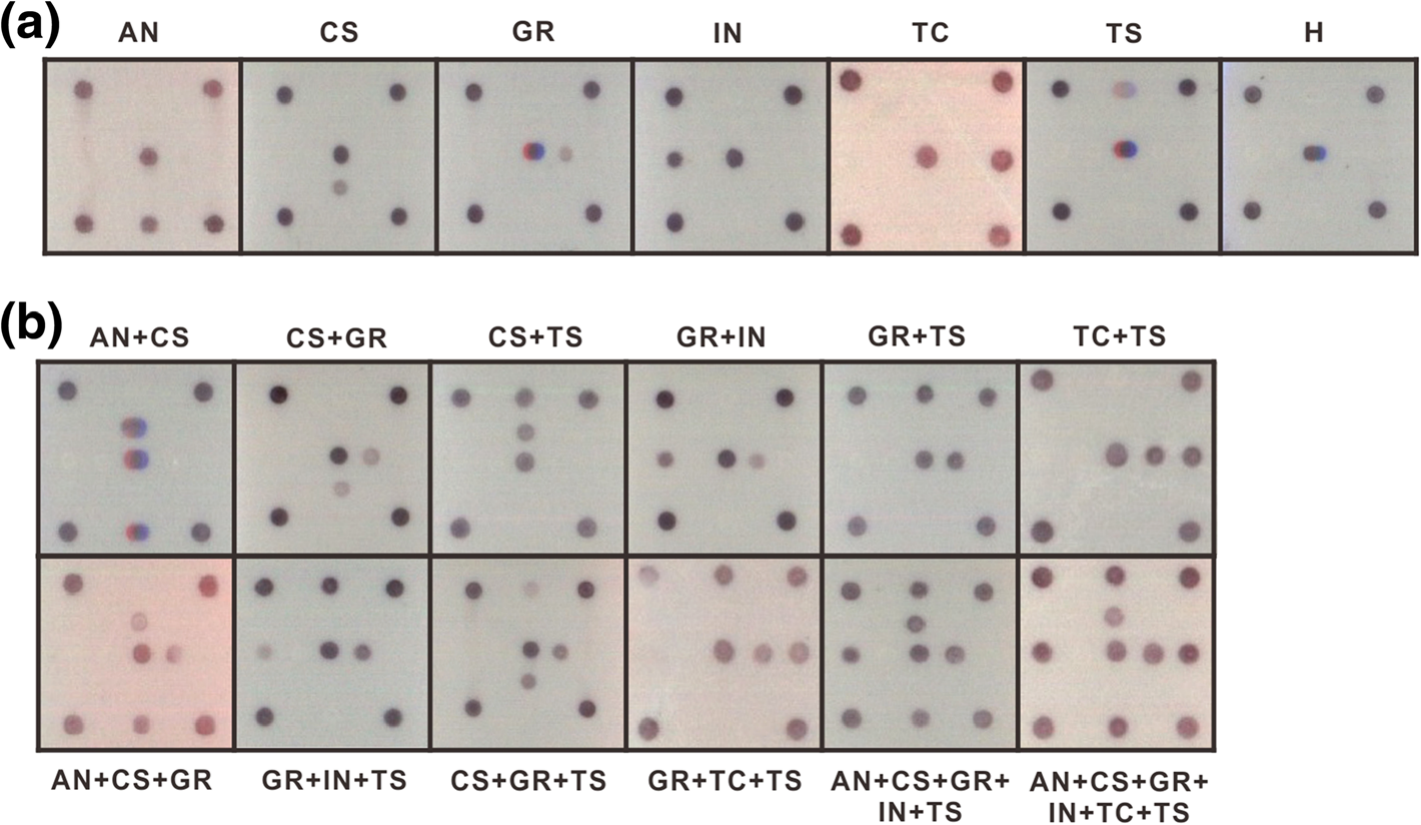
Microarray for detection of different tospoviruses in infected plant tissues. **a** Single virus detection. **b** Multiple virus detection. Total RNAs extracted from *Chenopodium quinoa* leaves separately inoculated with ANSV (AN), CSNV (CS), GRSV (GR), INSV (IN), TCSV (TC) and TSWV (TS) were used for tests. The total RNA of a healthy *C. quinoa* leaf (H) was used as the plant control. The degenerate primer pair Pr-dTS-f/Pr-dTS-r was used in a reverse transcription-polymerase chain reaction for amplification of N gene fragments from all tested tospoviruses. The positions of virus-specific probes spotted on the surface of biochips are represented in Fig. 2b

RT-PCR was performed to amplify the DNA fragment of 0.46 kb from the individual total RNAs of *C. quinoa* leaves separately inoculated with ANSV, CSNV, GRSV, INSV, TCSV or TSWV as well as the plasmid results shown in Fig. 1b. The amplicons were incubated with probe-spotted microchips mentioned in Fig. 2b, and all tested tospoviruses could be singly detected by the microarray (Fig. 4a). Furthermore, the total RNAs of different virus infections were mixed in 2–6 combinations for microarray assays, and individual tospovirus species could be identified (Fig. 4b). Our results demonstrated that the degenerate primer pair is sufficient for detection of the members of the TSWV serogroup and that the microarray is efficient in virus identification.

### Application of microarray for virus diagnosis in crop samples

**Fig. 5:**
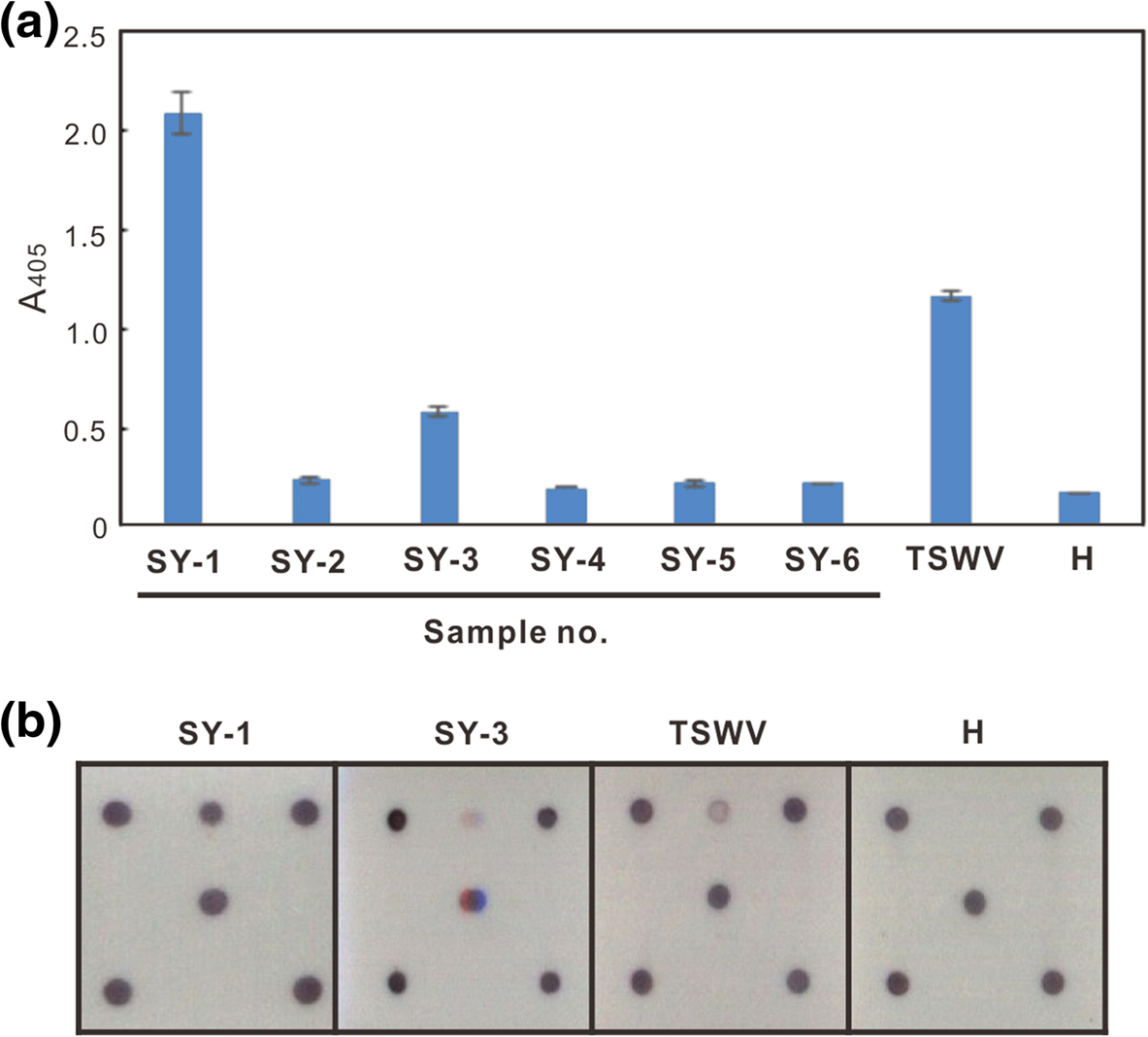
Application of the microarray for the diagnosis of tospovirus infections in field crop samples. **a** An indirect enzyme-linked immunosorbent assay was performed for detection of *Tomato spotted wilt virus* (TSWV) using the monoclonal antibody against the TSWV NSs protein, MAb-TNSs-82D3B4 [38]. **b** The microarray was conducted to detect the TSWV infection. The sample numbers of pepper samples collected from Sinyi Township of Nantou County, Taiwan are indicated as SY-1 to 6. The TSWV-infected *Chenopodium quinoa* sample was used as the positive control. The healthy *C. quinoa* leaf (H) was used as the negative control. Because SY-2, 4, 5 and 6 exhibited the identical results as well as the H control in the microarray assay, only the H result is shown

The diseased pepper samples (SY-1 to 6), exhibiting chlorotic spots or ringspots on leaves and fruits, were collected from Sinyi Township, Nantou County, Taiwan in December 2015 for detecting tospovirus infection by indirect ELISA using the monoclonal antibody against the TSWV NSs protein [39]. SY-1 and 3 exhibited an ELISA-positive (Fig. 5a). These samples were also used for assay in the microarray, and the identical result as well as indirect ELISA was obtained (Fig. 5b).

## Discussion

An efficient detection method is necessary for inspection of viral diseases in quarantine. The previous studies reported that the tospoviruses ANSV, CSNV, GRSV, INSV, TCSV and TSWV share a serological relationship in their NPs, so that they can be detected, but not be differentiated, by antisera against the NP in serological assays [28, 40]. On the other hand, several consensus sequences can be found among tospoviral genomic sequences. Degenerate primers designed from the consensus nucleotide sequences of RdRp and NSm genes have been used to detect tospoviruses at the genus level [30, 31]. The virus strains with different genotypes will not be excluded when degenerate primers are used in amplification. By contrast, the N genes of tospoviruses are more diverse, thus it can be the key target for classification of a tospovirus [6].

Consensus nucleotide sequences can also be found within the N genes of tospoviruses belonging to a phylogenetical clade. According to this finding, the degenerate primer pair Pr-dTS-f/Pr-dTS-r were designed from the N genes of all nine TSWV-serogroup tospoviruses, and can successfully detect all six tested viruses, ANSV, CSNV, GRSV, INSV, TCSV and TSWV, in one-step RT-PCR (Fig. 1). Unfortunately, the other three TSWV-serogroup viruses, MeSMV, PNSV and ZLCV, can not be obtained for test in the present study. Sequence comparison of the N genes of all members of TSWV serogroup was conducted to reveal that INSV shares the lowest nucleotide identity (59.3–61.7%) with other tospoviruses (Additional file 1: Table S1) and still can be detected by the degenerate primer pair Pr-dTS-f/Pr-dTS-r. Thus, the detection of MeSMV, PNSV and ZLCV using the degenerate primer pair Pr-dTS-f/Pr-dTS-r can be expected even if the virus materials are unavailable. The use of a universal primer pair in simplex RT-PCR significantly increases the efficiency of nucleic acid amplification by comparison with the use of combinations of various primer pairs in multiplex RT-PCR. Here, we first integrated the degenerate primers and specific probes into a microarray system to simultaneously detect and differentiate the TSWV-serogroup tospoviruses that possess twofold higher sensitivity than conventional RT-PCR (Fig. 3). The tospoviruses belonging to other serogroups, including Calla lily chlorotic spot virus (CCSV), Capsicum chlorosis virus (CaCV), *Groundnut bud necrosis virus* (GBNV), Groundnut chlorotic fan-spot virus (GCFSV), IYSV, Melon yellow spot virus (MYSV), Soybean vein necrosis-associated virus (SVNaV), Tomato necrotic spot-associated virus (TNSaV), Tomato yellow ring virus (TYRV), Tomato zonate spot virus (TZSV), *Watermelon bud necrosis virus* (WBNV) and WSMoV, were also used to test the developed microarray. No unexpected signals were observed to demonstrate the excellent specificity of the microarray in TSWV serogroup only (data not shown). Using the same approach, microarrays for detecting tospoviruses in other serogroups will be further developed.

The DNA microarray is basically developed as a combination technique of nucleic acid amplification and hybridization for high-throughput assay and has a wide application including use in analyses of gene expression, transcription factor binding and genotyping [41]. It also provides a benefit for simultaneous detection of numerous plant RNA viruses [32]. The reliability and time-and-cost-saving are very important issues in development of detection methods. In this study, we used the PVC biochip-based microarray platform developed by the Dr. Chip Company. Different from the common glass-slide microarray system, the signals on PVC biochips can be directly observed by the naked eye and recorded by an optical scanner or camera after colorization instead of the need of expensive equipment for fluorescent detection. It also provides an advantage of customized probe spotting. The reliability of the PVC biochip has been proven in several applications; e.g., the simultaneous detection and differentiation of seven mastitis-causing pathogens in bovine milk samples [37], Newcastle disease and avian influenza in the poultry industry [42], and *Cymbidium mosaic virus*, *Odontoglossum ringspot virus* and CaCV for orchid inspection (Dr. Orchid-3™ Kit, Dr. Chip), and the identification of insect species [43] and the sex of owls [44].

The developed microarray was used to inspect virus infection in the fields of Sinyi Township in central Taiwan, and indirect ELISA using MAb-TNSs-82D3B4 was performed for comparison. MAb-TNSs-82D3B4 reacted with TSWV but not reacted with GRSV, INSV and TCSV was mentioned in our previous report [39], and its serological reactivity with ANSV and CSNV was proven by our laboratory later. Thus, in this case, the ELISA-positive result might be resulted from the infections of ANSV, CSNV and/or TSWV (Fig. 5a). The developed microarray helps to clarify the result of indirect ELISA, indicating that two pepper samples, SY-1 and 3, exhibiting ringspots on leaves and fruits, were infected with TSWV only (Fig. 5b). No other TSWV-serogroup tospoviruses, such as ANSV, CSNV, GRSV and INSV, were found in Taiwan yet. Our results also demonstrated the reliability of the microarray in diagnosis of tospoviral diseases.

Tospoviruses are vectored by thrips for oversea distribution. The identification of the thrips category and the inspection of viruliferous thrips are necessary for preventing an invasion of foreign tospoviruses. The microarray is sufficient for multiplex detections. The identifications of tospoviruses and thrips will be integrated into a biochip to strengthen inspection for quarantine.

## Conclusions

In this study, a degenerate primer pair Pr-dTS-f/Pr-dTS-r, designed from the consensus sequences of N genes, was used to amplify a DNA fragment from total RNAs of plant tissues infected by six tested tospoviruses, ANSV, CSNV, GRSV, INSV, TCSV and TSWV. The virus species were further differentiated by hybridization with specific probes spotted on PVC biochips. The microarray platform was used to diagnose TSWV infections in field peppers. Our results showed that the microarray method can be used to simultaneously detect and identify TSWV-serogroup tospoviruses that provides a great applicable potential in inspection of tospoviruses for quarantine.

## Additional file

Additional file 1: Table S1. Nucleotide (above diagonal) and amino acid (below diagonal) identities (%) of the N genes among the members of TSWV serogroup. (DOCX 68 kb)

## Abbreviations

ANSV: Alstroemeria necrotic streak virus
CaCV: Capsicum chlorosis virus
CCSV: Calla lily chlorotic spot virus
CSNV: Chrysanthemum stem necrosis virus
ELISA: Enzyme-linked immunosorbent assay
GBNV: *Groundnut bud necrosis virus*
GCFSV: Groundnut chlorotic fan-spot virus
GRSV: *Groundnut ringspot virus*
GYSV: *Groundnut yellow spot virus*
INSV: *Impatiens necrotic spot virus*
IYSV: *Iris yellow spot virus*
MAb: Monoclonal antibody
MeSMV: Melon severe mosaic virus
MYSV: Melon yellow spot virus
NP: Nucleocapsid protein
ORF: Open reading frame
PNSV: Pepper necrotic spot virus
RT-PCR: Reverse transcription-polymerase chain reaction
SVNaV: Soybean vein necrosis-associated virus
TCSV: *Tomato chlorotic spot virus*
TNSaV: Tomato necrotic spot-associated virus
TSWV: *Tomato spotted wilt virus*
TYRV: Tomato yellow ring virus
TZSV: Tomato zonate spot virus
WBNV: *Watermelon bud necrosis virus*
WSMoV: *Watermelon silver mottle virus*
ZLCV: *Zucchini lethal chlorosis virus*
