# Supplementary material for: Development of a microarray for simultaneous detection and differentiation of different tospoviruses that are serologically related to Tomato spotted wilt virus
Source: Virol J. 2017 Jan 10;14:1. doi: 10.1186/s12985-016-0669-1 (PMC5234141; doi:10.1186/s12985-016-0669-1)
Supplement: Additional file 1: Table S1. — Nucleotide (above diagonal) and amino acid (below diagonal) identities (%) of the N genes among the members of TSWV serogroup. (DOCX 68 kb) [file 12985_2016_669_MOESM1_ESM.docx]

|  | ANSV | CSNV | GRSV | INSV | MeSMV | PNSV | TCSV | TSWV | ZLCV |
| --- | --- | --- | --- | --- | --- | --- | --- | --- | --- |
| ANSV | 100 | 75.9 | 76.8 | 59.3 | 63.8 | 84.4 | 78.0 | 78.8 | 75.2 |
| CSNV | 74.2 | 100 | 73.4 | 60.2 | 67.0 | 76.1 | 74.1 | 75.6 | 77.0 |
| GRSV | 81.8 | 72.7 | 100 | 60.9 | 64.3 | 79.2 | 82.6 | 77.0 | 74.8 |
| INSV | 54.2 | 54.5 | 54.4 | 100 | 60.1 | 60.0 | 61.7 | 59.4 | 59.7 |
| MeSMV | 58.8 | 60.3 | 58.8 | 51.3 | 100 | 64.8 | 63.7 | 62.6 | 65.9 |
| PNSV | 87.6 | 75.4 | 85.7 | 53.4 | 61.5 | 100 | 80.7 | 80.6 | 76.2 |
| TCSV | 81.8 | 74.2 | 87.6 | 54.9 | 59.5 | 84.5 | 100 | 77.3 | 74.3 |
| TSWV | 79.5 | 76.5 | 79.8 | 54.6 | 56.5 | 81.4 | 80.2 | 100 | 72.7 |
| ZLCV | 75.8 | 79.6 | 75.8 | 51.9 | 59.2 | 77.7 | 75.4 | 73.5 | 100 |

Supplementary Table 1. Nucleotide (above diagonal) and amino acid (below diagonal) identities (%) of the N genes among the members of TSWV serogroup

* The abbreviations of tospoviruses and the accession codes of their N gene sequences used for comparison are listed in Table 1.

** The ALIGN program of Biology WorkBech ver. 3.2 was used for analyses.
